# Use of Offer Bypass Filters under the Circular Kidney Allocation System

**DOI:** 10.34067/KID.0000000000000423

**Published:** 2024-04-03

**Authors:** Miko Yu, Kristen L. King, S. Ali Husain, Jesse D. Schold, Sumit Mohan

**Affiliations:** 1 Division of Nephrology, Department of Medicine, Columbia University Medical Center, New York, New York; 2 Columbia University Renal Epidemiology Group, New York, New York; 3 Department of Surgery, University of Colorado – Anschutz Medical Campus, Aurora, Colorado; 4 Department of Epidemiology, School of Public Health, University of Colorado – Anschutz Medical Campus, Aurora, Colorado; 5 Department of Epidemiology, Mailman School of Public Health, Columbia University, New York, New York

**Keywords:** kidney transplantation

In the United States, kidney transplant centers set organ offer filters, referred to by United Network for Organ Sharing as kidney minimum acceptance criteria filters, to allow candidates at their center to be automatically bypassed for offers from donors with prespecified characteristics to expedite allocation. We previously reported inefficiencies in the use of these filters, where centers with more restrictive filter settings did not display higher offer acceptance ratios despite prescreening out more offers.^1^ Interest in optimizing bypass filter usage has increased after implementation of the new distance-based kidney allocation system (KAS250) with the associated overwhelming increases in the volume of organ offers received.^2^ We, therefore, sought to determine whether centers have modified their bypass filter settings after the implementation of KAS250 to help mitigate the operational burden of responding to these additional offers.

Using data from United Network for Organ Sharing, we compared center-level bypass filter settings pre-KAS250 (January 2021), early post-KAS250 (April 2021), and 1 year later (April 2022), excluding pediatric centers (*n*=38) and centers performing no deceased donor kidney transplants in 2021/2022 (*n*=38) or with no filter settings recorded (*n*=10). Filter openness scores were calculated as the total number of filters set open to accepting organs from donors of that category or with settings more open/accepting than the January 2021 median across centers for continuous values (*e.g*., maximum donor age).^1^ We used Scientific Registry of Transplant Recipients kidney offer data to calculate the change in each transplant center's offer volume, defined using the total number of donors offered to a center with a response logged in each of the 3 months of interest. After dropping bypasses for reasons that were not under the transplant center's control, we calculated the center's proportion of candidate-level declined offers that were automatic filter-related bypasses (refusal codes 883 and 886) versus requiring a manual no response. We calculated Pearson correlations between change in filter openness score and each of these measures.

Among 192 included centers, each responded to a median 223 offers (interquartile range [IQR], 176–267) pre-KAS250 versus 266 (IQR, 207–307) offers early post-KAS250 and 349 (IQR, 289–403) offers 1 year later, corresponding to median increases in offer volume of 18% (IQR, 8%–29%) and 33% (IQR, 23%–42%) 1 month and 1 year after KAS250, respectively. Despite the increase in offer volume, centers had a median of 0% change (IQR, 0%–0%) in filter openness, with a median of 66 (IQR, 56–74) of 91 filters set open to receiving offers pre-KAS250 versus 66 (IQR, 55–75) early post-KAS250 and 68 (IQR, 57–75) 1 year later. There was no meaningful correlation between center-level change in filter openness and change in organ offer volume or between greater filter openness and their proportion of declined offers that were automatic bypasses in the early post-KAS250 period (*r*=−0.1889, *P* = 0.0090) versus 1 year later (*r*=−0.1305, *P* = 0.0728) (Figure 1). Changes in offer volume were larger between April 2022 and April 2021 than between April 2021 and January 2021, and changes varied across geographic regions (Supplemental Table 1). Most filter change records were submitted in May 2021 (Figure 1E).

**Figure 1:**
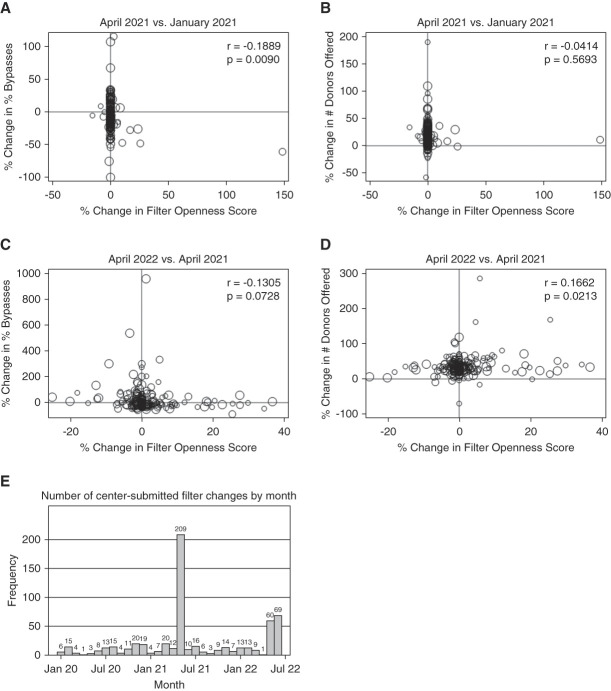
**Comparison of center-level changes in the early post-KAS250 period and 1 year later**. Center-level change in the proportion of offers that are declined offers and the change in the number of donors offers to centers as a function over changes in the center filter openness score between April 2021 and January 2021 (A and B) and April 2022 and April 2021 (C and D). Also shown is the frequency of center-submitted filter changes by month (E). These center filters are known as KiMAC filters by UNOS. KiMAC, kidney minimum acceptance criteria; UNOS, United Network for Organ Sharing.

Our results suggest that centers experienced a marked increase in organ offer volume after the introduction of KAS250 coupled with an increase associated with rising organ procurement but failed to modify their center-wide bypass filter settings to help reduce the proportion of offers that require manual responses.^3^ The reasons for the failure to change filter settings is unclear at present. While the fear of missing out on organ offers could be a driving factor, there may also be logistical concerns such as the administrative burden associated with the frequent review of these filters. Given that intracenter risk thresholds are also known to vary, settings may reflect the acceptance thresholds of some but not all of the clinicians in a program.

Optimizing the use of filters in the allocation process will need this process to be data driven and be informed by (*1*) an indicator of what proportion of the donor pool would be excluded with any given filter, (*2*) center-level information on current center-level practice to help inform how to set any given filter, and (*3*) transparent information on how these choices affect transplant probability for patients.

Policy proposals aimed at optimizing filter use through default filter settings are now under consideration but need urgent study before their implementation.^4^ Having center filter settings revert to the defaults reflective of recent practice risks the creation of a two-tier system based on center aggressiveness and thereby two levels of access to transplant for waitlisted candidates. For example, centers that have filters selected by default but do not experience a subsequent increase in organ offer acceptance rates risk having additional offers filtered, further decreasing the available pool of organs for their candidates. Preventing this unintended consequence will require the use of long intervals of data on which the defaults are based, allowing centers to review these changes annually and ensuring that patients and referring providers have access to information that allows them to decipher the implications of these choices. In the absence of this transparency, we are already seeing variations in the willingness to use kidneys from donors with hepatitis C, as identified by the use of the corresponding filter creating two tiers of centers—those that willing to consider these organs for their candidates and those that are not.^5^

Together with evidence of increasing cold ischemia time and organ discard, our findings suggests critical worsening of allocation system efficiency under KAS250.^6–8^ A major redesign of bypass filters may be needed to help centers manage the increased logistical burden under KAS250 while also overcoming the fear of missing out.

## Supplementary Material

SUPPLEMENTARY MATERIAL

## Data Availability

Partial restrictions to the data and/or materials apply. This study used data from the Scientific Registry of Transplant Recipients (SRTR). Data used in this study may be requested through a Data Use Agreement with SRTR.

## Supplemental Material

Supplemental Table 1. Changes in offer volume, filter openness score, and percent bypasses by transplant center size and geographic region across the United States
