# Supplementary material for: Use of Offer Bypass Filters under the Circular Kidney Allocation System
Source: Kidney360. 2024 Apr 3;5(5):756–8. doi: 10.34067/KID.0000000000000423 (PMC11146644; doi:10.34067/KID.0000000000000423)
Supplement: SUPPLEMENTARY MATERIAL [file kidney360-5-756-s001.pdf]

ASN American Society of Nephrology
JASN Journal of the American Society of Nephrology
KIDNEY360 Accessing Our World From Every Angle
CJASN Clinical Journal of the American Society of Nephrology

ASN Journal Disclosure Form

As per ASN journal policy, I have disclosed any financial relationship or commitment held by myself and/or my spouse/partner in the past 36 months as included below. I have listed my Current Employer below to indicate there is a relationship requiring disclosure. If no relationship exists, my Current Employer is not listed.

S. Husain reports the following:
Employer: Columbia University Medical Center

I understand that the information above will be published within the journal article, if accepted, and that failure to comply and/or to accurately and completely report the potential financial conflicts of interest could lead to the following: 1) Prior to publication, article rejection, or 2) Post-publication, sanctions ranging from, but not limited to, issuing a correction, reporting the inaccurate information to the authors' institution, banning authors from submitting work to ASN journals for varying lengths of time, and/or retraction of the published work.

Name: Syed Ali Husain
Manuscript ID: K360-2023-000786R1
Manuscript Title: Use of offer bypass filters under the updated kidney allocation system
Date of Completion: March 4, 2024
Disclosure Updated Date: November 29, 2023

ASN Journal Disclosure Form

As per ASN journal policy, I have disclosed any financial relationship or commitment held by myself and/or my spouse/partner in the past 36 months as included below. I have listed my Current Employer below to indicate there is a relationship requiring disclosure. If no relationship exists, my Current Employer is not listed.

K. King has nothing to disclose.

I understand that the information above will be published within the journal article, if accepted, and that failure to comply and/or to accurately and completely report the potential financial conflicts of interest could lead to the following: 1) Prior to publication, article rejection, or 2) Post-publication, sanctions ranging from, but not limited to, issuing a correction, reporting the inaccurate information to the authors' institution, banning authors from submitting work to ASN journals for varying lengths of time, and/or retraction of the published work.

Name: Kristen L. King
Manuscript ID: K360-2023-000786R1
Manuscript Title: Use of offer bypass filters under the updated kidney allocation system
Date of Completion: March 6, 2024
Disclosure Updated Date: January 9, 2024

ASN
American Society of Nephrology
JASN
Journal of the American Society of Nephrology
KIDNEY360
Accessing Our World From Every Angle
CJASN
Clinical Journal of the American Society of Nephrology

ASN Journal Disclosure Form

As per ASN journal policy, I have disclosed any financial relationship or commitment held by myself and/or my spouse/partner in the past 36 months as included below. I have listed my Current Employer below to indicate there is a relationship requiring disclosure. If no relationship exists, my Current Employer is not listed.

S. Mohan reports the following:
Employer: Columbia University; Consultancy: HSAG, Sanofi, Specialist Direct; Patents or Royalties: Columbia University; Advisory or Leadership Role: Deputy Editor, Kidney International Reports (ISN); Chair, UNOS, Data advisory committee, ; Member, SRTR Review Committee, ; Member, ASN Quality committee; National Faculty Chair, ETCLC; and Other Interests or Relationships: Research Funding from NIH (NIDDK, NIHMD and NIBIB) and Kidney Transplant Collaborative).

I understand that the information above will be published within the journal article, if accepted, and that failure to comply and/or to accurately and completely report the potential financial conflicts of interest could lead to the following: 1) Prior to publication, article rejection, or 2) Post-publication, sanctions ranging from, but not limited to, issuing a correction, reporting the inaccurate information to the authors' institution, banning authors from submitting work to ASN journals for varying lengths of time, and/or retraction of the published work.

Name: Sumit Mohan
Manuscript ID: K360-2023-000786R1
Manuscript Title: Use of offer bypass filters under the updated kidney allocation system
Date of Completion: January 22, 2024
Disclosure Updated Date: November 24, 2023

ASN Journal Disclosure Form

As per ASN journal policy, I have disclosed any financial relationship or commitment held by myself and/or my spouse/partner in the past 36 months as included below. I have listed my Current Employer below to indicate there is a relationship requiring disclosure. If no relationship exists, my Current Employer is not listed.

J. Schold reports the following:
Employer: University of Colorado; Consultancy: Sanofi Corporation, Novartis, Veloxis and eGenesis; Research Funding: One Legacy Foundation, NIH/NIDDK, Kidney Transplant Collaborative, Department of Defense, National Institutes of Health; Honoraria: Sanofi Inc, eGenesis; Advisory or Leadership Role: Data Safety Monitoring Board Member - Bristol Myers Squibb; ; Vice Chair UNOS Data Advisory Committee; UNOS Policy Oversight Committee; and Speakers Bureau: Sanofi.

I understand that the information above will be published within the journal article, if accepted, and that failure to comply and/or to accurately and completely report the potential financial conflicts of interest could lead to the following: 1) Prior to publication, article rejection, or 2) Post-publication, sanctions ranging from, but not limited to, issuing a correction, reporting the inaccurate information to the authors' institution, banning authors from submitting work to ASN journals for varying lengths of time, and/or retraction of the published work.

Name: Jesse D. Schold
Manuscript ID: K360-2023-000786R1
Manuscript Title: Use of offer bypass filters under the updated kidney allocation system
Date of Completion: March 4, 2024
Disclosure Updated Date: March 4, 2024

ASN
American Society of Nephrology
JASN
Journal of the American Society of Nephrology
KIDNEY360
Accessing Our World From Every Angle
CJASN
Clinical Journal of the American Society of Nephrology

ASN Journal Disclosure Form

As per ASN journal policy, I have disclosed any financial relationship or commitment held by myself and/or my spouse/partner in the past 36 months as included below. I have listed my Current Employer below to indicate there is a relationship requiring disclosure. If no relationship exists, my Current Employer is not listed.

M. Yu has nothing to disclose.

I understand that the information above will be published within the journal article, if accepted, and that failure to comply and/or to accurately and completely report the potential financial conflicts of interest could lead to the following: 1) Prior to publication, article rejection, or 2) Post-publication, sanctions ranging from, but not limited to, issuing a correction, reporting the inaccurate information to the authors' institution, banning authors from submitting work to ASN journals for varying lengths of time, and/or retraction of the published work.

Name: Miko Yu
Manuscript ID: K360-2023-000786R1
Manuscript Title: Use of offer bypass filters under the updated kidney allocation system
Date of Completion: March 4, 2024
Disclosure Updated Date: May 24, 2023
