# Supplementary material for: Use of Offer Bypass Filters under the Circular Kidney Allocation System
Source: Kidney360. 2024 Apr 3;5(5):756–8. doi: 10.34067/KID.0000000000000423 (PMC11146644; doi:10.34067/KID.0000000000000423)
Supplement: SUPPLEMENTARY MATERIAL [file kidney360-5-756-s002.pdf]

Supplemental Table 1. Changes in offer volume, filter openness score and percent bypasses by transplant center size and geographic region across the US.

	Median (IQR) Percent Change Across Centers					
	Donors Offered		Filter Openness Score		Percent Bypasses	
	Apr 2021 vs. Jan 2021	Apr 2022 vs. Apr 2021	Apr 2021 vs. Jan 2021	Apr 2022 vs. Apr 2021	Apr 2021 vs. Jan 2021	Apr 2022 vs. Apr 2021
All Centers	18 (8 - 29)	33 (23 - 42)	0 (0 - 0)	0 (-1 - 3)	-10 (-20 - 5)	-2 (-18 - 30)
Center Size						
Small	18 (7 - 29)	36 (24 - 52)	0 (0 - 0)	0 (0 - 6)	-7 (-21 - 5)	-4 (-21 - 15)
Medium	17 (9 - 26)	35 (25 - 44)	0 (0 - 0)	0 (-2 - 1)	-11 (-22 - -2)	0 (-18 - 41)
Large	18 (7 - 29)	27 (22 - 34)	0 (0 - 0)	0 (-2 - 1)	-9 (-19 - 14)	0 (-18 - 41)
US Region						
Northeast	26 (20 - 34)	29 (20 - 40)	0 (0 - 0)	0 (-2 - 0)	-18 (-28 - -11)	13 (-5 - 76)
Southeast	15 (8 - 20)	33 (25 - 40)	0 (0 - 0)	0 (-1 - 5)	-9 (-21 - 7)	-5 (-16 - 30)
Midwest	19 (11 - 36)	32 (23 - 41)	0 (0 - 0)	0 (0 - 3)	-11 (-19 - -6)	0 (-19 - 26)
Southwest	11 (6 - 19)	33 (21 - 41)	0 (0 - 0)	0 (-1 - 1)	0 (-10 - 13)	-10 (-23 - 0)
West	3 (-1 - 9)	41 (32 - 55)	0 (0 - 0)	0 (-2 - 3)	13 (3 - 25)	-13 (-21 - 8)
